# Primary and Nested PCR Amplification of B1 Gene to Confirm Seropositivity of Toxoplasmosis Among Cancer Patients in Sri Lanka

**DOI:** 10.1155/jotm/5040196

**Published:** 2025-02-27

**Authors:** G. P. C. Weerasooriya, A. Manamperi, B. M. H. A. Banneheke

**Affiliations:** ^1^Department of Parasitology, Faculty of Medical Sciences, University of Sri Jayewardenepura, Nugegoda, Sri Lanka; ^2^Molecular Medicine Unit, Faculty of Medicine, University of Kelaniya, Colombo, Sri Lanka; ^3^North Wales Medical School, Bangor University, Bangor LL57 2DG, UK

**Keywords:** cancer, immunocompromised, nested PCR, PCR, seropositivity, toxoplasmosis

## Abstract

Toxoplasmosis, caused by the parasite *Toxoplasma gondii*, affects approximately 30% of the human population worldwide. Reactivation of latent infection in immunocompromised people leads to fatal disease. This study aimed to determine the seropositivity of toxoplasmosis by enzyme-linked immunosorbent assay (ELISA), confirmed by primary polymerase chain reaction (PCR) and nested PCR (nPCR), and validate ELISA against nPCR among a group of cancer patients. Of the 321 participants, six (1.9%) patients were positive for nPCR and both IgG and IgM ELISA tests while 36 (11.2%) and 131 (40.8%) showed evidence of possibly acute and past infection, respectively, and 15 (4.7%) were indeterminate. Among them, 19 (5.9%) nPCR positives would have been ignored as having evidence of past infection. Four (1.2%) patients would not have been treated at all if only ELISA had been performed as they had indeterminate ELISA results. This is the first study that used primary and nPCR with B1 gene amplification for confirmation of toxoplasmosis among cancer patients in Sri Lanka. These findings emphasize the need for confirmatory tests, such as nPCR, particularly in cancer patients who exhibit a weak antibody response. Implementing such tests can aid clinicians in effectively managing these patients, given the rising incidence and mortality rates of cancer in Sri Lanka.

## 1. Introduction

Toxoplasmosis, a preventable and treatable infection, is caused by the parasite *Toxoplasma gondii* (*T. gondii)*. Members of the family Felidae including cats are the definitive hosts of *T. gondii* while warm-blooded animals including humans are intermediate hosts [1]. The modes of transmission are animal to human (zoonotic), foodborne, mother to child (congenital), and less frequent via organ transplantation or transfusion of blood from an infected donor. Less commonly, laboratory workers who handle infected blood with live tachyzoites can acquire infection by accidental inoculation [2].

Approximately 30% of the global population is infected with *T. gondii*, and the prevalence in humans differs among different countries and regions [3]. Symptoms of an infected person depend on the status of their immune system. The majority of immunocompetent individuals are asymptomatic or develop a mild flu-like illness with lymphadenopathy [4]. The parasites can persist and reactivate when the immune system is weakened specially in immunocompromised individuals causing life-threatening diseases. Parasite mainly infect the brain as a commonset site, followed by the eye, lung, bone marrow, peritoneum, and heart [5]. Involvement of the liver, kidney, pancreas, and spinal cord has also been reported rarely [5, 6]. Toxoplasmosis among cancer patients has been associated with Hodgkin's lymphoma, leukemia, myeloma, melanoma, and malignancies in the brain such as meningioma, astrocytoma, glioblastoma, and ganglioglioma [7, 8].

In healthy people, the immune sensors recognize infected *T .gondii* and induce the immune responses to limit the spread of parasites within the body. As a result of the recognition of the infection, the innate immune system works in the first line while the adaptive immunity system is involved in the long-term control of *T. gondii* [9]. The innate immune system recognizes the pathogen-associated molecular patterns (PAMPs) of *T .gondii* by pattern receptors (PRRs) like Toll-like receptors (TLRs) [10]. As a result of that, cytokines such as tumor necrosis factor alpha (TNF-α), interleukin-6 (IL-6), and IL-12 produce from the activated macrophages and dendritic cells [11]. The cytokine interferon-gamma (IFNγ) produced with the help of interleukin 12 (IL-12) also helps to control the parasite *T .gondii*. Furthermore, parasite replication is restricted by the production of IFN by natural killer (NK) cells activated by IL-12 [12]. Furthermore, indoleamine 2,3-dioxygenase (IDO) stimulated by IFN-γ also suppresses the growth of *T. gondii* in glioma cells, retinoblastoma cells, human fibroblast, and macrophages [9]. Chemokines such as C-C motif chemokine ligand 2 (CCL2) and C-X-C motif chemokine ligand 2 (CXCL2) are also produced as a defense mechanism, and they recruit monocytes and neutrophils to the infected sites [13]. In adaptive immunity, CD4+ T-cells (helper T-cells) support the immune response by releasing cytokines, while CD8+ T-cells (cytotoxic T lymphocytes) directly destroy infected cells. As immunocompromised patients such as cancer patients have weakened immunity systems compared to healthy people, the production of cytokines such as IFN-γ is reduced and weakens the ability to control the *T. gondii* parasite. These patients lose the function of adaptive immunity, due to the reduction of effectiveness in CD4+ and CD8+ T-cells [14]. Therefore, immunocompromised patients such as cancer patients are more vulnerable to these parasitic diseases.

The diagnosis of toxoplasmosis can be established by serology, by direct visualization of the parasite in clinical specimens, or by specific nucleic acid amplification by polymerase chain reaction (PCR) assay. The main and frequently used method of detection of toxoplasmosis is serology. In immunocompromised patients, *Toxoplasma* antibodies fail to rise, and thus serological tests may be less useful. In such instances, the detection of parasitic DNA using molecular techniques that have high sensitivity and specificity serves as an essential component in the diagnostic algorithm [6]. To detect parasitic DNA, PCR-based techniques have been developed to amplify the most conserved gene sequences among different strains of *T. gondii* including the B1 gene repetitive sequence, the P30 (SAG1) gene, *T. gondii* surface antigen 2 (SAG2) loci, and ribosomal DNA [15].

In this study, the B1 gene was amplified using PCR to detect parasitic DNA as *T. gondii*; B1 gene detection is superior as the gene has multicopy targets. This B1 gene has a high specificity in *T. gondii* and has been repeated 35 times in the genome [16]. Therefore, it is more sensitive in detecting *T. gondii* than single-copy targets such as *P30* [17].

The aim of this study was to determine seropositivity, confirm toxoplasmosis by molecular techniques, and validate enzyme-linked immunosorbent assay (ELISA) results against molecular technique test results. This is the first study that used primary and nested PCR (nPCR) with B1 gene amplification to diagnose toxoplasmosis among cancer patients (or any other category of immunocompromised patients) in Sri Lanka. As the incidence and mortality rates of malignancies are on the rise in Sri Lanka [18], this study is beneficial to clinicians when managing immunocompromised patients.

## 2. Method

### 2.1. Study Design, Duration, Location, Participant Criteria, and Ethical Approval

This descriptive cross-sectional study was conducted among a group (*n* = 321) of 18–80 years (male and female) adult patients with histologically proven malignancies, who are on any type of immunosuppressive anticancer therapies like chemotherapy, steroids, and radiotherapy, admitted to the medical wards at National Cancer Institute (Apeksha Hospital), Maharagama, Colombo, Sri Lanka, from November 2021 to March 2022. Patients who had newly diagnosed cancers but were not histologically confirmed, not undergoing immunosuppressive therapy, and those unable to communicate or understand were excluded from the study. Data collection and sample collection were done daily from all eligible inward patients. After obtaining informed written consent that was available in trilingual format (Sinhala, English, or Tamil), an interviewer-administered questionnaire was used to collect sociodemographic information such as data on risk factors (presence of cats, consumption of raw meat, hygienic practices, etc.), clinical manifestations of toxoplasmosis, and medical history of the malignancy.

Ethical approval was obtained from the Ethics Review Committee of the University of Sri Jayewardenepura (ref no: 13/21) and the Ministry of Health, Sri Lanka (ref no: ETR/AC/M3/38/2021). Administrative approvals were obtained from the director of the National Cancer Institute (Apeksha Hospital), Maharagama, in Colombo, Western Province, of Sri Lanka. Data were anonymized and stored maintaining confidentiality with access only for the members of the research group. Laboratory work was conducted at the Department of Parasitology, Faculty of Medical Sciences, University of Sri Jayewardenepura, Nugegoda, in Sri Lanka. Each patient was provided with a report that included a clear interpretation of the laboratory results, which was then forwarded to the attending physician to support proper patient management.

## 3. Collection of Blood Samples

Five milliliters of venous blood was collected from each patient: 2 mL into a plain tube for serology and the remaining 3 mL into ethylenediaminetetraacetic acid (EDTA) containing tube for DNA extraction by a trained nursing officer.

## 4. Serological Diagnosis

Blood samples in plain tubes were centrifuged at 3000 rpm for 15 min, separated sera were stored at −20°C, and within two weeks, anti-*T. gondii* IgG and IgM antibodies were tested using commercially available ELISA kits (CALBIOTECH, El Cajon, CA, USA) following the manufacturer's instructions. Two hundred microliters of sample diluent was added to 10 μL of each sample to prepare 1:21 dilutions of test samples. Hundred microliters of positive control, negative control, serum diluent, calibrator, and diluted samples was included in duplicates on every plate. According to the ELISA results, the patients were categorized into four groups, negative, with possible acute infection (IgM and IgG positive, IgM positive and IgG negative, IgM positive and IgG borderline, IgM borderline and IgG negative), with possible past infection (IgM negative and IgG positive, IgM borderline and IgG positive), and indeterminate (IgM negative and IgG borderline) [19].

## 5. Molecular Analysis

3 mL of blood collected from the EDTA tube was used for genomic DNA extraction on the same day of the collection using a QIAamp blood mini kit (Qiagen, Germany) for PCR. Extracted DNA was stored at −80°C for not more than one week to conduct Primary PCR. A 200-bp region of the *T. gondii* B1 gene was amplified by primary PCR in a 25-μl reaction mixture with GoTaq® green master mixture (Promega, USA). The required primers were prepared using already designed sequences (IDT, USA). The forward outer primer sequence was (OB1/F) (5′-GGAACTGCATCCGTTCATGAG-3′), and the reverse outer primer was (OB1/R) (5′-TCTTTAAAGCGTTCGTGGTC-3′). The primary PCR product was reamplified by nPCR with forward primer (IB1/F) (5′-TGCATAGGTTGCAGTCACTG-3′) and reverse primer (IB1/F) (5′-GGCGACCAATCTGCGAATACACC-3′) to obtain a final product of a one hundred base pair fragment [20]. Positive (*Toxoplasma gondii* RH 50174D, ATCC, USA) and negative (distilled water) controls were used in each batch of the PCR. A hundred-base pair ladder (GeneDirex, Taiwan) was used to detect DNA bands in gel electrophoresis.

## 6. Statistical Analysis

Data were analyzed and the receiver operating characteristic (ROC) curve was obtained to validate ELISA results against nPCR using the R Version 4.2.2 statistical program.

## 7. Results

### 7.1. Sociodemography of the Population

**Figure 1:**
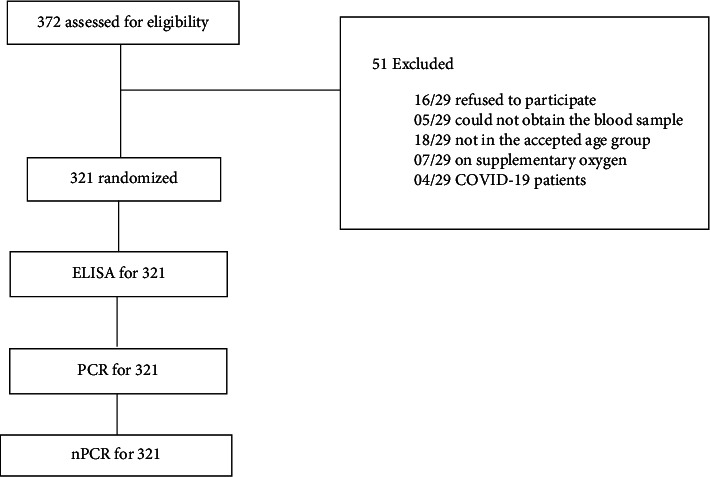
Consort diagram for the patients screened, randomized, and completed during follow-up.

**Figure 2:**
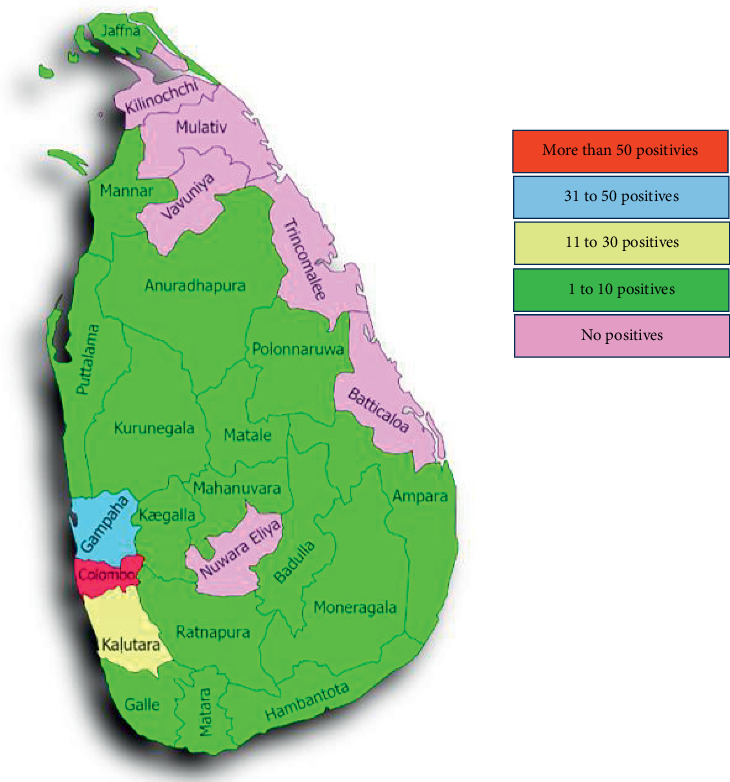
Distribution of seropositives (IgM or/and IgG positives) among the districts in Sri Lanka.

The total sample of 321 cancer patients (Figure 1) had equal gender distribution (females: 161, 50%, and males: 160, 50%). Their distribution in the nine provinces of Sri Lanka (Figure 2) was as follows: Western—185 (57.6%), Southern—32 (10%), Sabaragamuwa—29 (09%), North-Western—28 (8.7%), Uva—17 (5.3%), Central—11 (3.4%), North-Central—8 (2.5%), Eastern—6 (1.9%), and Northern—5 (1.6%). The mean age of the patients was 53.4 (SD = 16.6). The age distribution in the 18–40, 41–60, and 61–80 age groups was 82 (25.6%), 105 (32.7%), and 134 (41.7%), respectively. Among the study group, 191 (60%) had hematological cancers, and 130 (40%) had organ cancers. The study group included 22 (6.9%) patients only with primary education and 299 (93.1%) with secondary education. The monthly income level of the study group was 108 (33.6%) with less than 20,000 or no income and 213 (66.4%) with 20,000 and above. Among them, 135 (42.1%) were cat owners and 186 (57.9%) were not. Two (0.6%) patients in the study group eat raw meat while others eat cooked meat or only vegetables. According to the reports analyzed, none of the patients had significant clinical manifestations of toxoplasmosis at the time of the interview, but there were 6 patients with lymphadenopathy and fever.

### 7.2. Toxoplasmosis Seropositivity by ELISA

Seropositivity of toxoplasmosis among cancer patients was 31 (9.7%) for IgM and 153 (47.7%) for IgG. The number of patients with positive, negative, and borderline IgM results was 31 (9.7%), 273 (85%), and 17 (5.3%), while that for IgG was 153 (47.7%), 154 (48%), and 14 (4.3%), respectively. Patients with no serological evidence of toxoplasmosis (both IgG and IgM negative) were 139 (43.3%).

### 7.3. Confirmation of Toxoplasmosis by Molecular Technique Biology Testing

**Figure 3:**
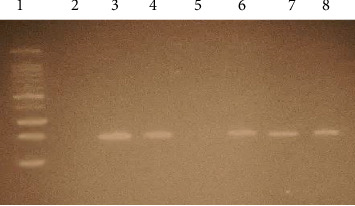
A gel image of primary PCR results of B1 gene amplification. Lane 1 is a 100 bp ladder, Lane 2 is negative control, Lane 3 is positive control, Lane 4 is toxoplasmosis primary PCR positive patient sample, Lane 5 is *T. gondii* primary PCR negative patient sample, and Lanes 6, 7, and 8 are toxoplasmosis primary PCR positive patient samples.

**Figure 4:**
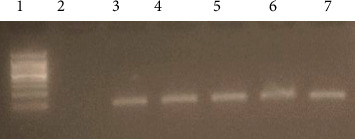
Gel image of toxoplasmosis nested PCR B1 gene amplification. Lane 1 is a 100 bp ladder, Lane 2 is a negative control, Lane 3 is a positive control, and Lanes 3, 4, 5, 6, and 7 are *T. gondii* nested PCR positive samples.

Thirty (9.3%) patients were positive for primary PCR, and all 30 were nPCR positive while 291/321 (90.7%) were negative.  Figure 3 shows an example of primary PCR results, and Figure 4 shows the nPCR results.

## 8. Comparison of ELISA and nPCR Results

A comparison of the ELISA and PCR results was done by adopting the categorization method by the Centers for Disease Control and Prevention, USA [19]. For ease of reference, they were categorized as A to D (Table 1).

## 9. Validation of ELISA Test Against nPCR

ELISA was validated against the nPCR. When only category “A” (see Table 1) [19] was evaluated, the sensitivity, specificity, positive predictive value (PPV), and negative predictive value (NPV) were 23.3%, 89.7%, 16.2%, and 91.5%. When categories “A” and “B” together were evaluated, the sensitivity, specificity, PPV, and NPV were 86.7%, 51.6%, 15.6%, and 97.4%, respectively.

## 10. ROC Curve for ELISA Test

**Figure 5:**
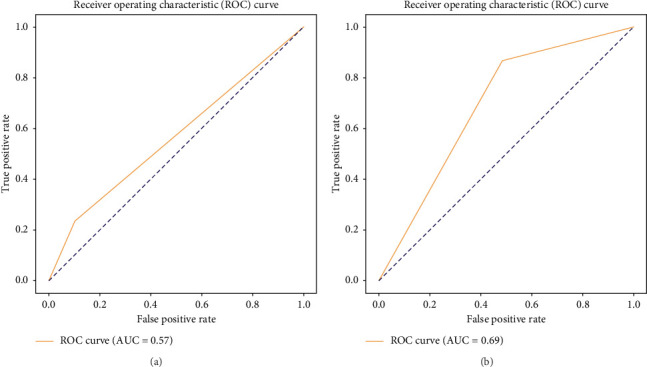
Receiver operating characteristic (ROC) curve of ELISA tests compared with gold standard nPCR results.

In the ROC curve, ELISA (category A alone) in comparison to the gold standard nPCR had an area under the curve (AUC) value of 0.57 (Figure 5(a)), while ELISA (categories A and B combined) with nPCR had an AUC of 0.69 (Figure 5(b)).

## 11. Discussion

Toxoplasmosis leads to complications among patients with malignancies due to compromised immunity due to the cancer itself and the type of treatments they receive. Therefore, screening cancer patients routinely for toxoplasmosis is worthwhile despite the presence or absence of symptoms. The present study focused on determining the prevalence of toxoplasmosis in the immunocompromised category of patients, taking a group of cancer patients as the example population. There are no published seropositivity or molecular studies done among cancer patients in Sri Lanka. The current study had equal distribution of male and female patients (females: 161, 50%, and males: 160, 50%) with mean age of 53.4 (SD = 16.6). At the time of sample collection, none of the patients showed specific toxoplasmosis symptoms, but six of the patients had lymphadenopathy and fever, and of these patients, 4 were nPCR-positive and 2 were negative. Among the 4 nPCR positives, 3 were ELISA negative and 1 was ELISA positive. Toxoplasmosis screening was conducted for the first time, as it was not previously part of the routine tests performed upon hospital admission. As a result, it is not possible to conclusively determine whether the detected infection represents reactivation.

A study done in Iran among 80 solid organ cancer patients and 33 hematological cancer patients showed that the prevalence of toxoplasmosis is higher among hematological cancer patients (41, 51.5%) than in those with solid organ cancers (11, 35%) [21]. In another study done among 106 patients with different types of cancer in Tehran, the only one patient who was PCR positive with higher IgG concentration had acute myeloid leukemia (AML) [22]. In the current study, 19 patients (52.8%) showed evidence of possibly acute toxoplasmosis and 23 patients (76.7%), who were PCR positive, had hematological cancers (Table 2). Fourteen (38.9%) of them with evidence of possibly acute infection and 10 (33.3%) of them with PCR results had AML. Higher prevalence of toxoplasmosis in hematological cancer patients such as AML patients can be due to weakened cellular immunity and inability to resist intracellular pathogen infections compared to other cancers [23]. Similarly, the evidence of elevated prevalence of toxoplasmosis among acute leukemia patients can be due to presence of immature myeloid cells which inhibits the antigen-specific T-cell responses [24].

Serological tests for the detection of IgM and IgG antibodies are the most common laboratory tests used for screening for toxoplasmosis, and ELISA format is often used by diagnostic laboratories. Both qualitative and quantitative ELISA kits are commercially available [25]. The qualitative ELISA tests cannot differentiate between acute and past infection. Even in quantitative format, a second blood sample in 3-4 weeks is recommended to see a four-fold rise in IgG antibody titer [26]. In the majority (73%) of toxoplasmosis patients, IgM antibodies appear 1 week after the infection, and antibody levels rise to peak after one to 3 months and disappear by decreasing over the next 9 months [25]. In a minority of 9 to 27% of patients, IgM antibodies persist for a lifetime [25]. Therefore, interpreting a positive IgM test result requires additional testing or clinical correlation for a more accurate diagnosis [27]. In the case of IgG antibodies, after 2 weeks of contracting the infection, IgG antibodies appear, increase to peak level within 3 months, remain at a constant level for 6 months, and over 1 year decrease to lower levels but persist for a lifetime [25]. In this study, based on ELISA results, 36 (11.2%) would have been suspected of having acute infection and treated. However, only seven (2.2%) were confirmed by nPCR tests. Among the study group, 6 (1.9%) patients were positive for PCR and both IgG and IgM ELISA tests (Table 1*).* Meanwhile, there were 19 (5.9%) nPCR positives that would have been ignored as having evidence of past infection. Four (1.2%) patients would not have been treated at all if only ELISA had been performed as they had indeterminate ELISA results. Therefore, these data will be beneficial to clinicians in detecting patients infected with *T. gondii* in this immunocompromised group and providing necessary treatment. A similar scenario has also been reported by other researchers. In a study done among 40 cancer patients (age 17–76 years) in India, 13 (32.5%) were positive for PCR which included two patients with positive IgG and IgM antibodies, nine with only IgG antibodies, and two with no antibodies by ELISA [28]. A study in Iran among 350 cancer patients reported nineteen (5.4%) PCR positives of which nine (2.6%) were IgM and IgG positive and ten (2.9%) were IgM negatives and IgG positive. In another study done in Egypt among 40 cancer patients, 30 (75%) were positive for IgG and three were positive for IgM by ELISA. In this study, PCR was positive in 13 (32.5%), of which, nine (22.5%) had IgG positive/IgM negative results while two (5%) were both IgG and IgM positive and two (5%) were both IgG and IgM negative [28]. A study among 35 cancer patients done in Iraq reported that 19 (54.2%) patients had both ELISA and PCR positive, while three (9%) of ELISA negatives had positive PCR [29].

Interpretation of ELISA results in toxoplasmosis is different and complicated compared to many other infections. Only IgM positive results could be due to false positivity [26]. In patients with only IgG-positive results in quantitative ELISA, to differentiate acute from past infection, a second sample should be tested for a four-fold rise after 3-4-week intervals [26]. That leads to practical difficulties due to the availability of patients for a second sample after 3-4 weeks. More importantly, it leads to delays in the commencement of treatment. Immunocompromised patients such as cancer patients are more vulnerable to these parasitic diseases [14]. The sensitivity and specificity of the ELISA in our study were 86.7% and 51.6%, respectively, which are comparable to the findings of a study from Iraq that reported a sensitivity of 54.2% and a specificity of 90% [29]. When ELISA test results were validated against nPCR considering only category A, the AUC was 0.57 which is only marginally better than a random guess. When the same is done for ELISA (categories A and B combined), the AUC value was 0.69 which is better than a random guess. That indicates that the performance of the ELISA is not accurate enough to be used as a specific test for toxoplasmosis specially to detect acute infection. In the primary PCR, two outer primers were used, while in nPCR, two inner primers were applied. This method minimizes the risk of false positives and false negatives [30].

In conclusion, the sensitivity of the ELISA test among this group of cancer patients was low, making it less suitable for screening for toxoplasmosis among cancer patients in Sri Lanka. The results of this study shed light on the importance of using a specific molecular technique test to confirm the routine serological test in cancer patients. This is very important to Sri Lanka where the incidence and mortality rates of malignancies are on the rise [18]. The same recommendation is applicable to countries facing similar challenges. The impact of sampling time is one of the drawbacks that could affect the results [31]. Furthermore, it would have been better if we could perform PCR immediately after DNA extraction, as it has more yield compared to stored DNA samples [32]. Another drawback of our study was the use of qualitative ELISA techniques due to restrictions in the budget. A quantitative ELISA would have provided more insight to the disease status [33]. The future direction of this research should extend to the use of quantitative screening of toxoplasmosis using quantitative ELISA and real-time PCR. This is a preliminary study carried out to screen toxoplasmosis among cancer patients in Sri Lanka, and a group of healthy population also will be screened in the next phase of the study.

## Figures and Tables

**Table 1:** Comparison of ELISA and PCR results.

Patient category based on serology⁣^∗^ and ELISA results *n* = 321 (%)	PCR results *n* = 321 (%)	
	PCR positive	PCR negative
• Category A: evidence of acute infection 36 (11.2)	7 (2.2)	29 (9.0)
IgM-positive and IgG-positive 18 (5.6)	06 (1.9)	12 (3.7)
IgM positive and IgG negative 12 (3.7)	0	12 (3.7)
IgM positive and IgG borderline 01 (0.3)	0	01 (0.3)
IgM borderline and IgG negative 05 (1.6)	01 (0.3)	04 (1.3)
• Category B: evidence of past infection 131 (40.8)	19 (5.9)	112 (34.9)
IgM negative and IgG positive 120 (37.4)	19 (5.9)	101 (31.5)
IgM borderline and IgG positive 11 (3.4)	0	11 (3.4)
• Category C: no evidence of toxoplasmosis 139 (43.3)	0	139 (43.4)
IgM negative and IgG negative 139 (43.3)	0	139 (43.3)
• Category D: indeterminate results 15 (4.7)	04 (1.2)	11 (3.4)
IgM negative and IgG borderline 15 (4.7)	04 (1.2)	11 (3.4)
IgM and IgG borderline 0 (0)	0 (0)	0 (0)

⁣^∗^Adapted from CDC [19].

**Table 2:** Significant association of cancer type versus ELISA and nPCR results.

Diagnosis	Indeterminate	Not infected	Possibly acute infection	Possibly past infection	PCR positive	PCR negative
Acute lymphoblastic leukemia (ALL)	1 (0.3%)	10 (3.1%)	2 (0.6%)	14 (4.4%)	1 (0.3%)	26 (8.1%)
Acute myeloid leukemia (AML)	5 (1.6%)	32 (9.9%)	14 (4.4%)	30 (9.3%)	12 (3.7%)	69 (21.6%)
Angiosarcoma	0	0	0	2 (0.6%)	0	2 (0.6%)
B-cell acute lymphoblastic leukemia (B-ALL)	0	4 (1.2%)	1 (0.3%)	3 (0.9%)	1 (0.3%)	7 (1.9%)
B-cell lymphoma (BCL)	0	2 (0.6%)	0	0	0	2 (0.6%)
^∗^CA anus	0	1 (0.3%)	0	0	0	1 (0.3%)
^∗^CA bladder	0	1 (0.3%)	0	1 (0.3%)	0	2 (0.6%)
^∗^CA breast	1 (0.3%)	12 (3.7%)	2 (0.6%)	8 (2.5%)	2 (0.6%)	21 (6.5%)
^∗^CA bronchi	0	1 (0.3%)	0	0	0	1 (0.3%)
^∗^CA cervix	0	4 (1.2%)	0	4 (1.2%)	0	8 (2.5%)
^∗^CA cheek	0	0	0	1 (0.3%)	0	1 (0.3%)
^∗^CA glottis	0	1 (0.3%)	1 (0.3%)	0	0	2 (0.6%)
^∗^CA colon	0	2 (0.6%)	2 (0.6%)	3 (0.9%)	1 (0.3%)	6 (1.9%)
^∗^CA colorectal	0	1 (0.3%)	0	0	0	1 (0.3%)
^∗^CA endometrium	0	2 (0.6%)	0	5 (1.6%)	0	7 (2.2%)
^∗^CA esophagus	0	1 (0.3%)	1 (0.3%)	5 (1.6%)	1 (0.3%)	6 (1.9%)
^∗^CA gastroesophageal junction (GOJ)	0	2 (0.6%)	0	1 (0.3%)	0	3 (0.9%)
^∗^CA gallbladder	0	1 (0.3%)	0	0	0	1 (0.3%)
^∗^CA gastric	0	1 (0.3%)	0	2 (0.6%)	0	3 (0.9%)
^∗^CA liver	0	0	0	1 (0.3%)	0	1 (0.3%)
^∗^CA lung	2 (0.6%)	5 (1.6%)	2 (0.6%)	2 (0.6%)	1 (0.3%)	10 (3.1%)
^∗^CA oral	0	1 (0.3%)	1 (0.3%)	2 (0.6%)	0	4 (1.2%)
^∗^CA ovary	0	0	0	1 (0.3%)	0	1 (0.3%)
^∗^CA penis	0	1 (0.3%)	0	0	0	1 (0.3%)
^∗^CA prostate	0	0	1 (0.3%)	1 (0.3%)	0	2 (0.6%)
^∗^CA recto sigmoidal	0	1 (0.3%)	1 (0.3%)	0	0	2 (0.6%)
^∗^CA rectum	0	4 (1.2%)	2 (0.6%)	1 (0.3%)	0	7 (2.2%)
^∗^CA squamous cell carcinoma (SCC)	0	0	1 (0.3%)	0	0	1 (0.3%)
^∗^CA spine	0	1 (0.3%)	0	0	0	1 (0.3%)
^∗^CA supraglottic	0	0	0	1 (0.3%)	0	1 (0.3%)
^∗^CA testis	0	1 (0.3%)	0	0	0	1 (0.3%)
^∗^CA thyroid	0	0	0	1 (0.3%)	0	1 (0.3%)
^∗^CA tonsil	0	0	0	1 (0.3%)	0	1 (0.3%)
^∗^CA tongue	0	0	1 (0.3%)	0	0	1 (0.3%)
Chronic granulocytic leukemia (CGL)	0	1 (0.3%)	0	0	0	1 (0.3%)
Chronic lymphocytic leukemia (CLL)	0	8 (2.5%)	0	6 (1.9%)	1 (0.3%)	13 (4.0%)
Chronic myeloid leukemia (CML)	0	0	1 (0.3%)	0	0	1 (0.3%)
Cutaneous T-cell lymphoma (CTCL)	1 (0.3%)	0	1 (0.3%)	0	1 (0.3%)	1 (0.3%)
Diffuse large B-cell lymphoma (DLBL)	1 (0.3%)	1 (0.3%)	0	3 (0.9%)	0	1 (0.3%)
Ewing sarcoma	0	4 (1.2%)	0	6 (1.8%)	1 (0.3%)	3 (0.9%)
Hairy cell leukemia (HCL)	0	0	0	1 (0.3%)	2 (0.6%)	8 (2.5%)
Hodgkin lymphoma (HL)	0	2 (0.6%)	0	0	0	1 (0.3%)
Hepatosplenic T-cell lymphoma (HSTCL)	1 (0.3%)	0	0	0	0	2 (0.6%)
Mixed phenotype acute leukemia (M-ALL)	0	1 (0.3%)	0	0	0	1 (0.3%)
Myelodysplastic syndrome (MDD)	0	1 (0.3%)	0	0	0	1 (0.3%)
Myelodysplastic syndrome (MDS)	1 (0.3%0)	8 (2.4%)	0	5 (1.5%)	0	1 (0.3%)
Mycosis fungoides (MF)	0	1 (0.3%)	0	0	0	14 (4.4%)
Multiple myeloma (MM)	0	1 (0.3%)	0	0	0	1 (0.3%)
Meningioma	0	0	0	1 (0.3%)	0	1 (0.3%)
Myelofibrosis	0	2 (0.6%)	2 (0.6%)	3 (0.9%)	0	1 (0.3%)
Non-Hodgkin lymphoma (NHL)	0	1 (0.3%	0	0	2 (0.6%)	5 (1.6%)
Neuroblastoma	1 (0.3%)	7 (2.1%)	0	10 (3%)	0	1 (0.3%)
Osteosarcoma	0	1 (0.3%)	0	0	2 (0.6%)	16 (5.0%)
Squamous cell carcinoma (SCC)	0	1 (0.3%)	0	0	0	1 (0.3%)
Spindle cell carcinoma (SPCC)	1 (0.3%)	5 (1.5%)	0	4 (1.2%)	0	1 (0.3%)
T-cell acute lymphoblastic leukemia (T-ALL)	0	0	0	1 (0.3%0)	2 (0.6%)	8 (2.5%)
T-cell non-Hodgkin lymphoma (TNHL)	0	1 (0.3%)	0	0	0	1 (0.3%)
T-cell lymphoma (TCL)	0	0	0	1 (0.3%)	0	1 (0.3%)
T-cell large granular lymphocytic leukemia (TLL)	0	1 (0.3%)	0	0	0	1 (0.3%)

*Note:p* values are used for diagnosis and ELISA; *p* = 0.824.

^∗^CA: carcinoma.

## Data Availability

Data can be provided on request; they are available from the authors.
