# Corrigendum to “Primary and Nested PCR Amplification of B1 Gene to Confirm Seropositivity of Toxoplasmosis Among Cancer Patients in Sri Lanka”

**DOI:** 10.1155/jotm/9827589

**Published:** 2025-09-11

G. P. C. Weerasooriya, A. Manamperi, and B. M. H. A. Banneheke, “Primary and Nested PCR Amplification of B1 Gene to Confirm Seropositivity of Toxoplasmosis Among Cancer Patients in Sri Lanka,” *Journal of Tropical Medicine* 2025 (2025): 5040196, https://doi.org/10.1155/jotm/5040196.

In the article titled “Primary and Nested PCR Amplification of B1 Gene to Confirm Seropositivity of Toxoplasmosis Among Cancer Patients in Sri Lanka”, there was an error in affiliation number 2. In the corrected affiliation, the “*Colombo*” should be changed to “*Ragama*” in the article. The corrected affiliation appears below:

“Molecular Medicine Unit, Faculty of Medicine, University of Kelaniya, Ragama, Sri Lanka”

We apologize for this error.

